# Differentiated transplant derived airway epithelial cell cytokine secretion is not regulated by cyclosporine

**DOI:** 10.1186/1465-9921-12-44

**Published:** 2011-04-10

**Authors:** Timothy Floreth, Eric Stern, Yingli Tu, Randi Stern, Edward R Garrity, Sangeeta M Bhorade, Steven R White

**Affiliations:** 1 Section of Pulmonary and Critical Care Medicine, The University of Chicago, Chicago, IL 60637, USA

## Abstract

**Background:**

While lung transplantation is an increasingly utilized therapy for advanced lung diseases, chronic rejection in the form of Bronchiolitis Obliterans Syndrome (BOS) continues to result in significant allograft dysfunction and patient mortality. Despite correlation of clinical events with eventual development of BOS, the causative pathophysiology remains unknown. Airway epithelial cells within the region of inflammation and fibrosis associated with BOS may have a participatory role.

**Methods:**

Transplant derived airway epithelial cells differentiated in air liquid interface culture were treated with IL-1β and/or cyclosporine, after which secretion of cytokines and growth factor and gene expression for markers of epithelial to mesenchymal transition were analyzed.

**Results:**

Secretion of IL-6, IL-8, and TNF-α, but not TGF-β1, was increased by IL-1β stimulation. In contrast to previous studies using epithelial cells grown in submersion culture, treatment of differentiated cells in ALI culture with cyclosporine did not elicit cytokine or growth factor secretion, and did not alter IL-6, IL-8, or TNF-α production in response to IL-1β treatment. Neither IL-1β nor cyclosporine elicited expression of markers of the epithelial to mesenchymal transition E-cadherin, EDN-fibronectin, and α-smooth muscle actin.

**Conclusion:**

Transplant derived differentiated airway epithelial cell IL-6, IL-8, and TNF-α secretion is not regulated by cyclosporine *in vitro*; these cells thus may participate in local inflammatory responses in the setting of immunosuppression. Further, treatment with IL-1β did not elicit gene expression of markers of epithelial to mesenchymal transition. These data present a model of differentiated airway epithelial cells that may be useful in understanding epithelial participation in airway inflammation and allograft rejection in lung transplantation.

## Background

Lung transplantation is an accepted therapeutic approach to selected end-stage lung diseases. Despite improvement in peri-operative and early post-transplant outcomes, lung transplant recipients do not obtain the equivalent allograft longevity and resultant survival conferred upon other solid organ recipients [1]. Long-term outcomes in lung transplantation have been complicated by chronic rejection in the form of Bronchiolitis Obliterans Syndrome (BOS) with 50% of patients affected at five years [2,3].

Clinical events that correlate with the eventual development of BOS include primary graft dysfunction, acute rejection, viral respiratory infections, and gastroesophageal reflux although the mechanisms by which these events contribute to BOS have not been discerned [4]. While the histopathology of BOS has been described, a complete understanding of the causative pathophysiology remains elusive. Early inflammatory lesions in BOS are characterized by bronchiolar epithelial invasion by mononuclear cells with marked neutrophilia. After resolution of inflammation, fibrosis of the epithelium and airway lumen become the dominant histopathology [5].

Murine tracheal transplantation models suggest that airway epithelial cells (AEC) are a target of immune mediated injury in BOS [6]. Sera from lung transplant recipients with BOS have been shown to contain increased HLA and non-HLA antibodies directed against AEC [7,8]. Binding of these HLA antibodies to AEC lines elicits production of fibrogenic growth factors with subsequent fibroblast proliferation, suggesting that airway epithelial cells may have a role in transforming an alloimmune signal into a fibrotic process [9]. This transformation from inflammation to fibrosis occurs at or near the epithelium and may be part of the pathology of BOS.

While research suggesting a role for AEC in the pathophysiology of BOS has focused primarily upon alloimmune processes, less attention has been directed toward the innate inflammatory response of the epithelium to the local dynamic environment. Unlike other transplanted solid organs, the pulmonary allograft and airway epithelium are exposed to 10,000 liters of environmental air and its contents daily [10]. The potential role of AEC to participate in and direct innate immunity through secretion of cytokines, such as IL-6, IL-8, and TNF-α, and growth factors such as TGF-β1, in response to this dynamic local milieu is well established, but the ability of these factors to participate in dysregulated inflammation in the setting of systemic immunosuppression and, thereby, contribute to the genesis of BOS has not been investigated in lung transplantation [11].

Previous investigation has demonstrated a differing impact of immunosuppressive agents upon AEC cytokine and growth factor secretion *in vitro*, depending upon the experimental approach [12-15]. One important agent is cyclosporine, a calcineurin inhibitor used in combination with other agents in lung transplantation. Airway epithelial cells express cyclophilin, the cytosolic receptor for cyclosporine, and treatment of primary AEC grown in submersion culture with cyclosporine leads to inhibition of proliferation and increases in IL-1β stimulated IL-8 release [13]. Other studies using alveolar and central airway epithelial cell lines [15] suggest that calcineurin inhibitors can up-regulate IL-6 and IL-8 production. However, the effect of calcineurin inhibitors on epithelial cell function may depend on the state of differentiation and presence of cell subtypes typically not present in submersion culture, such as ciliated and goblet cells.

The growth factor TGF-β1, a potent stimulator of lung fibroblast proliferation and extracellular matrix production [16] and differentiation into myofibroblasts [17], also can induce epithelial to mesenchymal transition (EMT) to a myofibroblast-like phenotype in human AEC, [16-18] as suggested by de novo or increased expression of tenascin C, alpha-smooth muscle actin (SMA) and EDN-fibronectin and concomitant decreased expression of the epithelial-specific marker E-cadherin [18]. This transformation may be a critical step in the process of obliterative bronchiolitis in chronic lung allograft rejection in a process similar to that seen in other fibrotic lung diseases such as idiopathic pulmonary fibrosis [19,20]. One prior study demonstrated the expression of EMT markers in epithelial cells collected by bronchoscopy from stable lung transplant recipients [21], suggesting the presence of EMT and airway remodeling is associated with the clinical presentation of BOS [22]. In addition, cytokines such as TNF-α have recently been shown to potentiate the effect of TGF-β1 towards EMT in epithelial cells [23-25]. Taken together, these data suggest that a certain milieu of cytokines and growth factors must be present to elicit EMT sufficient to cause pathological changes to airways.

We hypothesized that cyclosporine would alter the secretion of selected cytokines and growth factors, and potentially alter the process of EMT, in AEC collected from lung transplant recipients. To answer this question, we collected cells from lung transplant recipients by endobronchial brushing and grew these cells in air liquid interface (ALI) culture to force differentiation and the development of goblet and ciliated cells. Our data demonstrate that cyclosporine does not attenuate the secretory response of airway epithelial cells to a standard stimulus, IL-1β. These results suggest that cyclosporine in physiologic, non-toxic concentrations has little effect on secretion of cytokines and growth factors by differentiated AEC. In addition, neither IL-1β nor cyclosporine induced gene expression of markers characteristic of epithelial to mesenchymal transition. Cyclosporine does not regulate key cytokine secretory functions in differentiated AEC that are associated with BOS.

## Materials and methods

### Patients

The recruitment of lung transplant recipients and the use of primary human airway epithelial cells collected by bronchoscopy in these patients were approved by the University of Chicago Institutional Review Board. Patients were recruited for this study from the population of lung transplant recipients at the University of Chicago. Nine patients, age 25 to 64 years, participated through the period of the current study undergoing a total of 12 bronchoscopies. The indications for transplantation included idiopathic pulmonary fibrosis (N = 4), chronic obstructive pulmonary disease (N = 3, one with both IPF and COPD), and one patient each with cystic fibrosis, eosinophilic granuloma, and alpha-1 anti-trypsin deficiency. All patients were between 3 and 12 months post-transplant and were clinically stable undergoing outpatient surveillance bronchoscopy. Patients underwent standard immunosuppression per protocol, which did not include cyclosporine. Pathologic evaluation of transbronchial biopsies collected at the time of sampling was notable for three episodes of acute rejection with only one episode greater than A1. Additionally, culture of bronchoalveolar lavage collected at the time of sampling was notable for significant isolation of specific organisms in three patients, including *Mycobacterium avium intracellulare, Mycobacterium gordonae*, and *Pseudomonas aeruginosa*. Neither rejection nor isolation of an organism impacted the ability to culture airway epithelial cells over baseline.

### Bronchoscopy

Informed consent was obtained from each subject prior to participation. Conscious sedation was employed with midazolam and fentanyl, and vital signs were monitored throughout the procedure. After inspecting both lungs and the anastomoses, bronchoalveolar lavage was obtained from either the right middle lobe or lingula. Following this, two cytology brushings with a protected epithelial cell cytology brush (Medical Engineering Laboratory, Shelby, NC) were collected from subsegmental bronchi and immediately placed in Clonetics media consisting of Bronchial Epithelial Cell Basal Media and SingleQuots supplements and growth factors (Lonza, Walkersville MD). Transbronchial biopsies were then done for both clinical and research indications. All patients recovered uneventfully from bronchoscopy.

### Airway epithelial cell culture

We have previously described our cell culture methods [26]. Brushes were placed in supplemented Clonetics media and gently shaken. This media was set aside and then supplemented Clonetics was titrated against the cytology brushes to ensure maximal harvesting of epithelial cells. Both samples were then centrifuged for three minutes at 1500 rpm and pelleted. Pellets were then resuspended in 2 ml of supplemented Clonetics media with a final antimicrobial regimen consisting of 50 μg/ml amphotericin, 50 μg/ml gentamicin, 100 U/ml penicillin, and 100 μg/ml streptomycin and plated in collagen-IV coated T25 flasks. After two days a further 3 ml of supplemented Clonetics media was added. On day four, the media was changed and subsequently changed every two days until cells were 85% confluent. Cells were passed to collagen-IV coated T75 flasks for further expansion and then were transferred (passage 2) to 12-well transwell filter membranes (10^5^/well) coated with collagen-IV. Cells were grown in ALI media consisting of 1:1 supplemented Clonetics and DMEM (Mediatech, Manassas VA) supplemented with 50 nM retinoic acid, 130 mg/L bovine pituitary extract, and 50 ug/ml low-endotoxin BSA. Cells were fed both apically and basally every 48 hr until confluence was achieved. Cells then were transitioned to ALI conditions and were only fed through the transwell basal compartment with the apical compartment exposed to air. Cells were fed every 48 hr for three weeks.

### Demonstration of cell differentiation

We have previously described these methods [26]. To demonstrate cell differentiation in air liquid interface culture, immunofluoresence labeling and confocal microscopy were utilized. Epithelial cells in ALI culture × 3 wk were fixed with 4% paraformaldehyde and then stained with antibodies directed against cytokeratin-5 (CK-5, clone RCK103, Santa Cruz Biotechnology, Santa Cruz CA) marking basal cells, Mucin 5AC (clone C-20, Santa Cruz Biotechnology, Santa Cruz CA) binding goblet cells and β-tubulin (catalogue # ab6046, Abcam Inc., Cambridge, MA) marking ciliated cells. Epithelial cell purity was determined using an anti-vimentin (clone V9, ZYMED Laboratories, Carlsbad CA) antibody to detect contaminating fibroblasts, and an anti-CD68 antibody (clone KP1, Dako, Carpinteria CA) to detect contaminating alveolar macrophages with IMR-90 primary lung fibroblast cell line and cytospin preparations of bronchoalveolar lavage specimens as positive controls, respectively.

### Treatment with IL-1β and cyclosporine

All cells were at passage two and in ALI culture for at least 15 days prior to initiation of the experimental protocol. Interleukin-1β was selected as a stimulus to examine epithelial cell cytokine secretion as it elicits secretion of both IL-8 [19,20] and IL-6 [21,22] from cultured AEC. Experimental arms consisted of treatment with 10 ng/ml IL-1β (R and D Systems, Minneapolis MN) alone, 1000 ng/ml cyclosporine (Sigma-Aldrich, St. Louis MO) alone, both IL-1β and cyclosporine, or control vehicle (0.01% ethanol). Each intervention was tested separately in the apical and basal compartments and assayed in triplicate. Cells in the vehicle and cyclosporine arms were treated daily over the five-day protocol with appropriately supplemented culture media. The IL-1β arms were treated daily with media for the first four days and received IL-1β supplemented media on the fifth day. Cells receiving both IL-1β and cyclosporine were treated with media plus cyclosporine for the first four days and on the fifth day received media supplemented with both cyclosporine and IL-1β. On day 6 (21 days of ALI culture), samples were collected for assays. The apical side of the cell layer was washed with 200 μl of ALI medium. The conditioned media from the basal compartment was retrieved. The cell layers were harvested, washed, and pelleted. All samples were stored at -80°C until use.

### Quantification of cytokine and growth factor secretion

IL-6, IL-8, TNF-α and TGF-β1 concentrations in conditioned media from the basal compartment were determined via ELISA (R and D Systems, Minneapolis MN) for each factor following kit directions. Samples were diluted as required. Blank, non-conditioned ALI was assayed at the same time to ensure that detected IL-6, IL-8, TNF-α and TGF-β1 concentrations represented secretion from cells.

### Real-time reverse transcription-polymerase chain reaction

Total RNA was isolated from cells using a PerfectPure RNA 96 Cell Kit (5 Prime, Gaithersburg, MD) following the manufacturer's protocol. Samples were treated with DNase I (5 Prime). Total RNA was reverse transcribed using random primers and Superscript II reverse transcriptase (Invitrogen, Carlsbad, CA). Real-time RT-PCR was performed using a Bio-Rad iCycler iQ PCR Detection System using iQ Supermix (Bio-Rad, Hercules, CA), and gene-specific primers as listed in Table 1.

**Table 1:** Primers used for real-time RT-PCR

Gene	Forward	Reverse
E-cadherin	5'-CGGGAATGCAGTTGAGGATC-3'	5'-AGGATGGTGTAAGCGATGGC-3'

α-SMA	5'-CTGGCATCGTGCTGGACTCT-3'	5'- GATCTCGGCCAGCCAGATC-3'

EDA-FN	5'-GAGCTATTCCCTGCACCTGATG-3'	5'-CGTGCAAGGCAACCACACT-3'

TGF-β	5'-ACCGGCCTTTCCTGCTTCTCA-3'	5'-CGCCCGGGTTATGCTGGTTGT-3'

GAPDH	5'-AGCCACATCGCTCAGACACCA-3'	5'-GCAAATGAGCCCCAGCCTTC-3'

SMA, smooth muscle actin, FN, fibronectin, TGF, transforming growth factor

### Statistics

Cytokine secretion data are expressed as the mean ± SEM. Real-time RT-PCR data are expressed as fold-change from control using GAPDH as an internal standard. Differences in cytokine secretion were examined by analysis of variance; when significant differences were found, post-hoc analysis was done using Fisher's protected least significant difference test. Differences in gene expression from control were examined using the 95% confidence interval. Differences were considered significant when P < 0.05.

## Results

### Cell Culture and Differentiation

**Figure 1:**
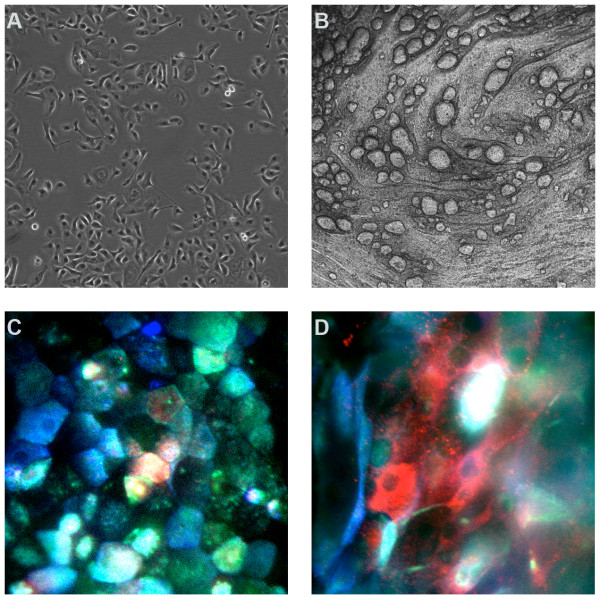
**Pulmonary allograft epithelial cells in culture**. A. Phase-contrast image of cells in submersion culture. B. Phase-contrast image of cells in air liquid interface culture for 3 weeks. C and D. Confocal microscopy of air liquid interface cells at 3 weeks. Cells were labeled with antibodies directed against ciliated cells (blue), goblet cells (red), or basal cells (green). White represents the overlap of all three colors and denotes an indeterminate cell. Original magnification of for A and B, 40 ×, and C and D, 400 ×.

Cells were collected from twelve bronchoscopies on nine patients. Eight of these from eight different patients yielded viable cells that were grown in submersion culture, successfully expanded, and then differentiated in ALI culture. All differentiated cells maintained cell layer integrity throughout the experimental protocol (Figure 1).

Labeling and confocal microscopy demonstrated the simultaneous presence of all three major AEC types: basal cells, goblet cells, and ciliated cells (Figure 1). Staining for CD68 was negative in cells at ALI demonstrating absence of macrophages. Staining for vimentin demonstrated less than 1% labeling in cells maintained in ALI cultures for 3 wk. Double staining techniques demonstrated that cells that labeled for vimentin did not label for CK-5, MUC5AC, or β-tubulin, thus suggesting minimal residual contamination with fibroblasts from the original collection.

### Secretion of IL-8

**Figure 2:**
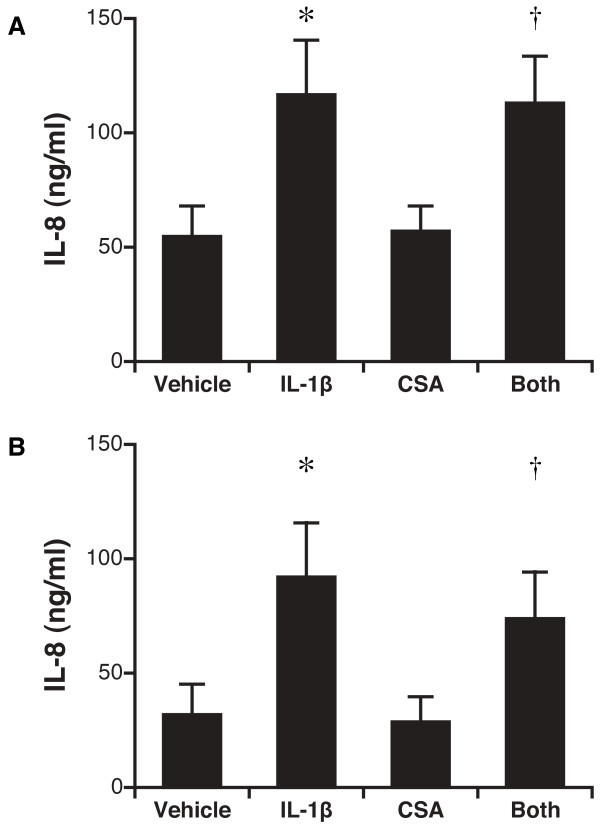
**Secretion of IL-8 by transplant-derived airway epithelial cells after stimulation with IL-1β and cyclosporine**. A. IL-8 secretion in basal medium after basal stimulation. *, P = 0.03 for IL-1β vs. control; †, P = 0.04 for IL-1β and cyclosporine vs. cyclosporine alone. B. IL-8 secretion in basal medium after apical stimulation. *, P = 0.006 for IL-1β vs. control; † P = 0.03 for IL-1β and cyclosporine vs. cyclosporine alone. N = 5 unique patient samples.

Stimulation with IL-1β in either the apical or basal compartment significantly up-regulated IL-8 secretion to the basal compartment: the concentration after basal IL-1β treatment was 117 ± 24 ng/ml (vs 55 ± 13 ng/ml for control, P = 0.03), whereas the concentration after apical IL-1β treatment was 92 ± 19 ng/ml (vs 32 ± 7 ng/ml for control, P = 0.01) (Figure 2). Treatment with cyclosporine in either the basal or apical compartment had no impact upon IL-8 secretion when compared with control vehicle, and further did not impact IL-1β stimulated differentiated airway epithelial cell secretion of IL-8 (Figure 2). In addition, there was no significant difference in IL-8 secretion either basal or apically between those treated with IL-1 β alone versus those with IL-1 β and cyclosporine (P = NS; Figure 2).

### Secretion of IL-6

**Figure 3:**
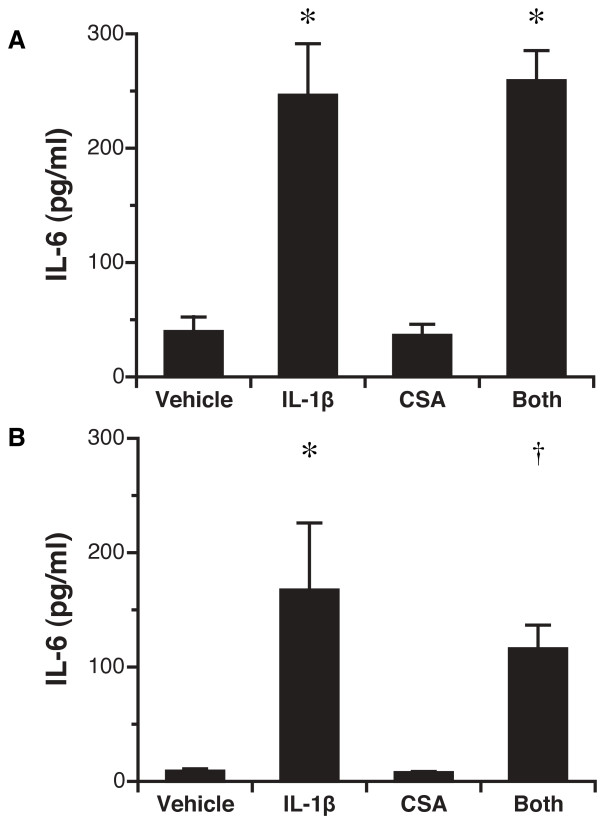
**Secretion of IL-6 by transplant-derived airway epithelial cells after stimulation with IL-1β and cyclosporine**. A. IL-6 secretion in basal medium after basal stimulation. *, P < 0.0001 for IL-1β vs. control and for IL-1β and cyclosporine vs. cyclosporine alone. B. IL-6 secretion in basal medium after apical stimulation. *, P = 0.002 for IL-1β vs. control; †, P = 0.02 for IL-1β and cyclosporine vs. cyclosporine alone. N = 5 unique patient samples.

As with IL-8, stimulation with IL-1β in either the apical or basal compartment significantly up-regulated IL-6 secretion in the basal compartment: the concentration with basal IL-1β treatment was 246 ± 45 pg/ml (vs 40 ± 12 pg/ml for control, P = 0.001), whereas the concentration with apical IL-1β treatment was 167 ± 58 pg/ml (vs 9.0 ± 4.4 pg/ml for control, P = 0.002) (Figure 3). As with IL-8, cyclosporine treatment altered neither baseline release nor IL-1β stimulated release of IL-6 (Figure 3). In addition, there was no significant difference in IL-6 secretion either basal or apically between those treated with IL-1 β alone versus those with IL-1 β and cyclosporine (P = NS; Figure 3).

### Secretion of TNF-α

**Figure 4:**
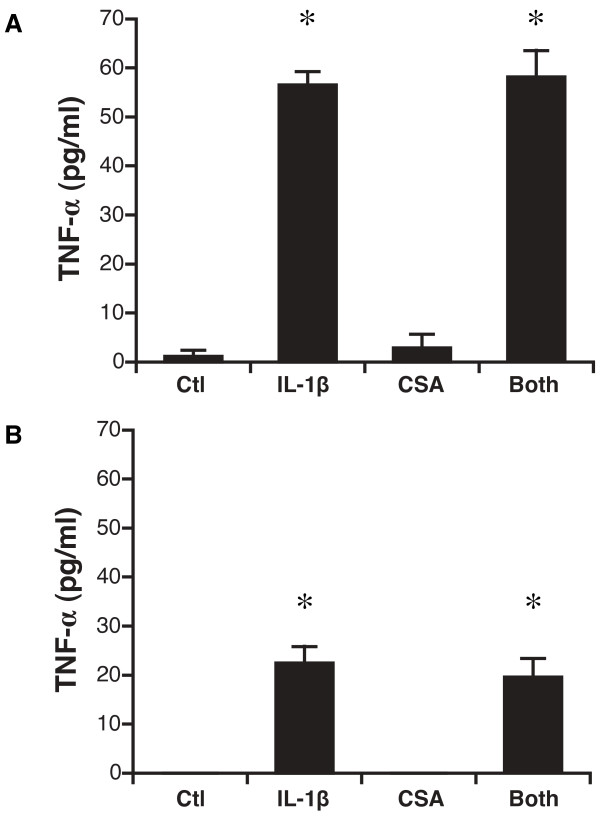
**Secretion of TNF-α by transplant-derived airway epithelial cells after stimulation with IL-1β and cyclosporine**. A. TNF-α secretion in basal medium after basal stimulation. *, P < 0.0001 for IL-1β vs. control and for IL-1β and cyclosporine vs. cyclosporine alone. B. TNF-α secretion in basal medium after apical stimulation. *, P = 0.0001 for IL-1β vs. control and for IL-1β and cyclosporine vs. cyclosporine alone. N = 5 unique patient samples except for apical stimulation with both IL-1β and cyclosporine, for which N = 3.

As with the other interleukins, stimulation with IL-1 β in either the apical or basal compartment significantly up-regulated secretion of TNF-α in the basal compartment: the concentration with basal IL-1 β treatment was 57 ± 6.1 pg/ml (vs 1.2 ± 2.6 pg/ml, P = 0.0001) and with apical IL-1β treatment was 22 ± 7.6 pg/ml (vs 0.0 ± 0.0 pg/ml for control, P = 0.0001) (Figure 4). As with IL-6 and IL-8, cyclosporine treatment altered neither baseline release nor IL-1β stimulated release of IL-6 (P = NS; Figure 4).

### Secretion of TGF-β1

TGF-β1 concentrations in cell-conditioned medium, for any experimental intervention, were not higher than that found in bland medium (data not shown).

### Gene expression of TGF-β

**Figure 5:**
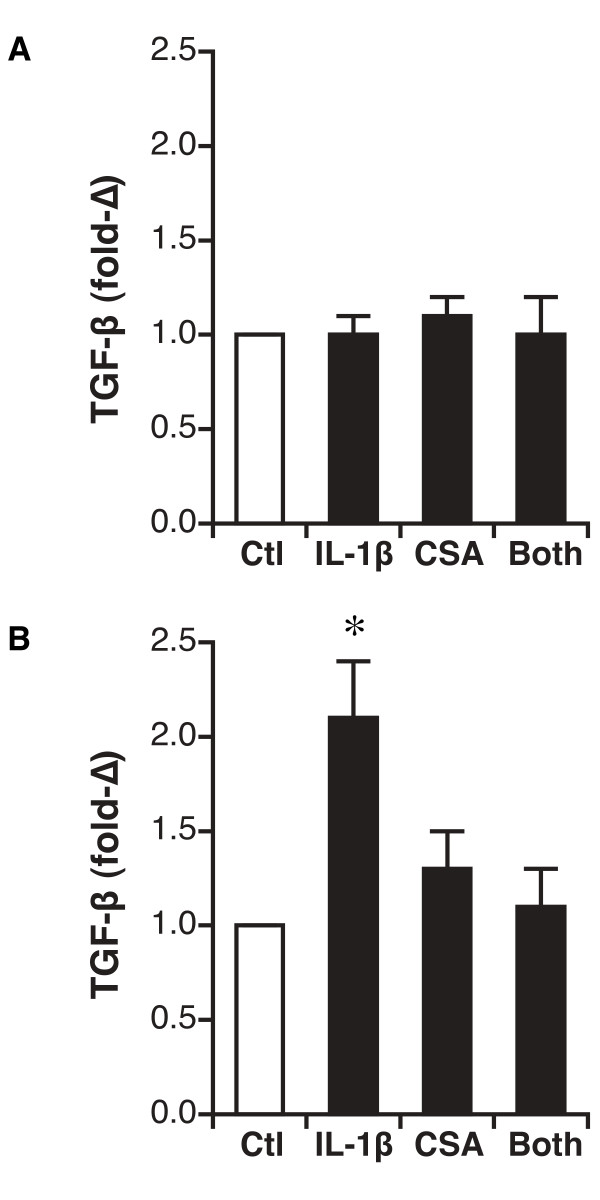
**Expression of TGF-β1 in transplant-derived differentiated airway epithelial cells**. Expression after either basal (Figure 5A) or apical (Figure 5B) addition of mediators is shown. N = 5 unique patient samples for each. *, P < 0.05 versus vehicle control. CSA, cyclosporine.

Expression of TGF-β also did not differ after treatment of differentiated AEC with IL-1β, cyclosporine or the combination over 24 hr when added to the basal compartment of the ALI culture (Figure 5). Addition of IL-1β to the apical compartment elicited a 2.1 ± 0.3 fold increase in TGF-β expression which was not seen when cells were treated with either cyclosporine alone or the combination of IL-1β and cyclosporine (Figure 5).

### Gene expression of EMT markers

**Figure 6:**
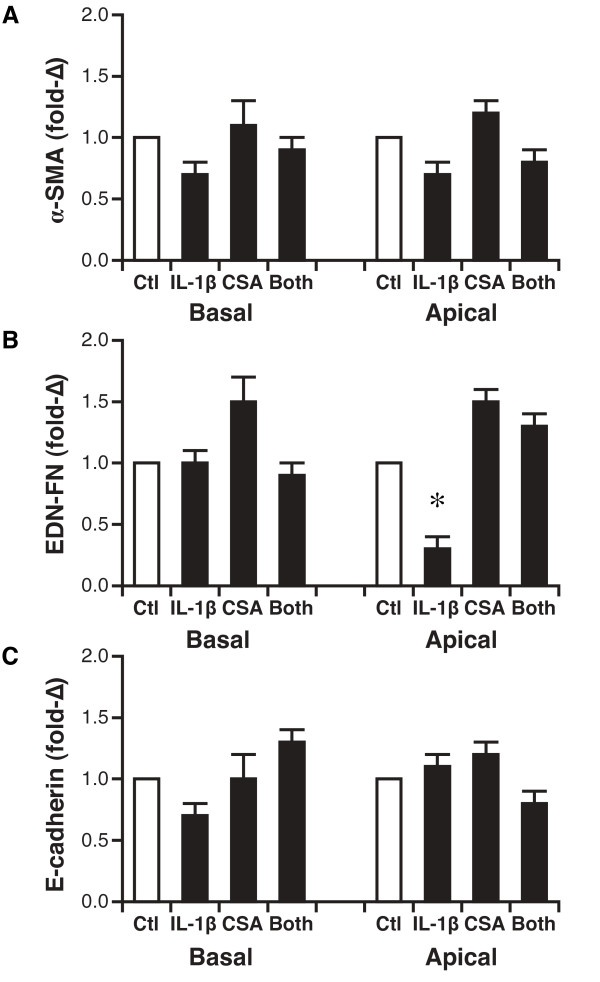
**Expression of markers of epithelial-mesenchymal transformation in transplant-derived differentiated airway epithelial cells**. Expression after either basal or apical addition of mediators is shown. A. Expression of α-smooth muscle actin (SMA). B. Expression of EDN-fibronectin. C. Expression of E-cadherin. N = 5 unique patient samples. *, P < 0.05 versus vehicle control. CSA, cyclosporine, SMA, smooth muscle actin, FN, fibronectin.

Expression of the myofibroblast markers α-smooth muscle actin (SMA) and EDN-fibronectin, and the epithelial cell marker E-cadherin, as measured by real-time RT-PCR following each experimental intervention to the basal compartment of the ALI culture was not different than that found in control, differentiated AEC (Figure 6). Addition of IL-1β to the apical compartment decreased EDN-fibronectin expression significantly; this was not seen when cells were treated with either cyclosporine alone or the combination of IL-1β and cyclosporine (Figure 6).

## Discussion

Long-term allograft survival in lung transplantation is limited by BOS, in which epithelial inflammation and fibrosis over time becomes a prominent component [3]. Epithelial cell secretion of chemotactic factors for neutrophils, such as IL-8 [27,28] and IL-6 [29], and pro-fibrotic factors such as TGF-β [27] and TNF-α, participate in overall small airway obliteration over time. In this study, we demonstrate that the potent immunosuppressive, cyclosporine, does not alter AEC secretion of IL-8, IL-6, TNF-α and TGF-β after stimulation with a known secretogogue for both IL-8 and IL-6, IL-1β, nor does it elicit expression of factors known to be associated with epithelial-mesenchymal transition. These data suggest that cyclosporine neither suppresses nor up-regulates processes critical to the genesis of BOS.

Of significant importance, we demonstrated that in contrast to studies using cells grown in submersion culture [12,13,15] cyclosporine did *not *increase IL-6 and IL-8 production in differentiated, transplant-derived airway epithelial cells. Airway epithelial cells have been shown to contain cyclophilin, the cytosolic receptor for cyclosporine, [13] and thus may regulate epithelial function in a manner similar to that seen in lymphocytes. The ability of AEC to respond to appropriate stimuli with production of inflammatory mediators despite cyclosporine administration suggests that even in immunosuppressed lung transplant patients, AEC may release inflammatory mediators in response to environmental stimulation without regulation by the immunosuppressive agent cyclosporine, and further suggests that mechanisms by which cyclosporine modulates the development of BOS does not include modulation of inflammatory factors secreted by AEC.

To the best of our knowledge, our study represents the first report of primary airway epithelial cells from lung transplant recipients grown in air liquid interface culture with resultant differentiation into mucous producing goblet, ciliated and basal cells. The use of ALI culture permits challenge to either the apical or basal cell layer surface. The apical (air exposed) surface, covered with goblet cell produced mucous, models the airway lumen while the matrix-coated filter approximates the basement membrane. Media supplied through the basal compartment delivers agents, nutrients, and potential irritants. This differentiation and polarity create a useful model of airway epithelium that differs from submersion culture techniques in which cells maintain a basal cell phenotype forming monolayers that can only be fed and challenged from a single cell surface.

Previous investigations utilizing non-transplant airway epithelial cells demonstrate that in the setting of immunosuppression, cells grown in ALI cell culture conditions respond to stimuli in a manner different than that seen in cells grown in submersion culture [13-15]. Given that differentiated cells must utilize resources and energy to maintain their unique phenotypes and interact with diverse surrounding cell populations, they may respond to stimuli in a different fashion than cell monolayers in submersion culture. In addition, not only do the cells present in differentiated culture differ in type and proportion but the environment in which each individual cell type must respond to stimuli differs as well.

Immunofluorescent labeling demonstrated the presence of all three cell types: ciliated, goblet, and basal. Control staining demonstrated the lack of macrophages as CD68+ labeled cells were not present after ALI culture × 3 wk. This is a useful advantage compared to cells grown in submersion culture, in which macrophages persisted up to at least passage 2 [30]. Further, few contaminating fibroblasts were demonstrated in ALI cultures, confirming observations of Forrest, et al, who previously demonstrated a lack of fibroblast contamination in transplant-derived epithelial cells grown in submersion culture [30]. The use of differentiated AEC devoid of other, contaminating cell types is a useful advantage to examine the response to immunosuppressive agents in isolation in AEC.

We demonstrated that differentiated airway epithelial cells collected from lung transplant recipients can respond to stimuli such as IL-1β to secrete cytokines such as IL-6, IL-8, and TNF-α, which then may modify further the local microenvironment. IL-8 is an important chemokine leading to neutrophil chemotaxis and IL-6 has been associated with early inflammation in the setting of tissue damage. TNF-α not only plays a central role inflammation but also in apoptosis. It has been shown that IL-1β may increase the number of TNF receptors, but the finding that this cytokine can induce TNF-α secretion in differentiated human AEC is also novel [31]. Each of these processes may be important in the pathogenesis of BOS. Our data suggest the possibility that airway epithelium may be more than just a target of injury in BOS but may participate in creating or perpetuating an inflammatory milieu at the interface between the lungs and the environment, the anatomic interface where BOS localizes.

One potential mediating role of the airway epithelium to injury and disordered repair in the pathogenesis of BOS may be stimulation of fibroblast proliferation, a process that can be mediated by growth factors such as TGF-β1. However, we were not able to demonstrate TGF-β1 secretion by differentiated transplant AEC even though gene expression was found to be increased when stimulated apically. A prior paper utilizing AEC lines showed only indirect evidence of fibrogenic growth factor secretion through utilization of blocking antibodies in fibroblast proliferation studies; actual cytokine and growth factor levels were not assayed [9]. Another limiting factor in our observations is that TGF-β1 has a short half-life in acidic environments and thus may not maintain structural integrity in conditioned media where the latent form has nothing to bind to inhibit rapid degradation [29].

One potential benefit of working with primary cells from lung transplant recipients is that patient outcomes, including early BOS, may be correlated with epithelial cell function. Although the proportional magnitude of response to stimuli appears similar, absolute quantities of cytokine production vary between patients, leaving open the possibility that patients whose epithelial cells produce higher levels of cytokines may be more prone to peribronchiolar inflammation and eventual BOS.

In our study, quantitative gene expression of α-SMA, EDN-fibronectin, and E-cadherin were substantially unchanged in response to IL-1β and/or cyclosporine, suggesting that neither the inflammatory cytokines added or produced by the AEC themselves nor the immunosuppressive agent shifted the phenotype of differentiated, transplant-derived AEC towards EMT. Indeed, apical treatment with IL-1β elicited a decrease, not increase, in EDN-FN (Figure 5), which would not be expected if epithelial cells were shifting to a mesenchymal phenotype. A prior study had noted changes even in asymptomatic transplant patients, but examined morphology of cells as a marker of EMT rather than gene expression [16]. Another study has demonstrated that EMT can occur in normal epithelial after stimulation with TGF-β1 [15]. The lack of TGF-β1 expression in our study may thereby explain the lack of EMT marker expression in differentiated AEC. Therein, a threshold dose or time above a threshold dose of TGF-β1 alone or in combination with other cytokines such as TNF-α may not have been met and thereby EMT may not have occurred. Lastly, other key cell types such as neutrophils and macrophages may need to be present in this milieu to elucidate EMT. Further studies are needed to delineate the process by which EMT occurs both ex-vivo and in-vivo and therein ways to interrupt it may allow treatment modalities in the future.

## Conclusions

In summary, we demonstrate IL-6, IL-8, and TNF-α secretion, but not TGF-β1 secretion, in response to IL-1β stimulation in differentiated AEC collected from stable lung transplant recipients. Secretion is not affected by treatment with cyclosporine in contrast to studies using cells grown in submersion culture. In addition, neither treatment with IL-1β nor cyclosporine induced gene expression that would be expected in epithelial-mesenchymal transformation. Our study suggests that transplant-derived AEC grown in differentiated culture have a response to cytokines different from that seen in similar cells grown in submersion culture. These responses may be useful in understanding the role of airway epithelium in processes associated with BOS and chronic allograft rejection.

## Competing interests

The authors declare that they have no competing interests.

## Authors' contributions

All authors have read and approved the final manuscript.

ES conceived the study, participated in the design and coordination of experiments and drafted the manuscript. TF completed final experiments and analysis, and edited the final manuscript. YT performed experiments and data analysis, and RS provided technical assistance. EG and SB performed bronchoscopy, provided cells from consented post transplant patients and assisted in conceptual design. SW provided mentorship, conceptual design, statistical analysis and final manuscript review.
